# Intravascular Ultrasound-Guided Stenting for Iatrogenic Aortocoronary Dissection: The Art of “Needlework”

**DOI:** 10.1016/j.cjco.2025.04.009

**Published:** 2025-04-20

**Authors:** Quentin Liabot, Jérémie Macia, Stéphane Fournier, Guillaume Testu de Balincourt, Aurelia Zimmerli, Marion Dupré, Victor Weerts, Olivier Muller, Adil Salihu, Louis Viallard, David Meier

**Affiliations:** a Cardiology Department, Lausanne University Hospital and University of Lausanne, Lausanne, Switzerland; b Cardiology Department, Henri Mondor Hospital Centre, Aurillac, France; c Cardiology Department, Clermont-Ferrand University Hospital, Clermont-Ferrand, France

Iatrogenic aortocoronary dissection (IACD) is a rare but serious complication of percutaneous coronary interventions (PCI).^1^ Wiring the true lumen (TL) is particularly challenging since the dissection extends up to the very ostium of the artery. Here, we describe the management of IACD using intravascular ultrasound (IVUS) to guide a double-wiring technique.

A 71-year-old female patient presented with an inferior ST-elevation myocardial infarction. Emergent angiography revealed acute occlusion of the right coronary artery (RCA). Unfortunately, during the intubation of a 6-F AL0.75 guiding catheter (Launcher guiding catheter, Medtronic, Minneapolis, MN) into the RCA ostium, repeated back-and-forth movements of the catheter (Video 1 , view video online), combined with small contrast injections, led to a type D dissection with secondary extension to the aortic root, precluding further contrast injections (Fig. 1A; Video 2 , view video online). Thus, IVUS was felt to be the only way to guide the TL wiring.

**Figure 1:**
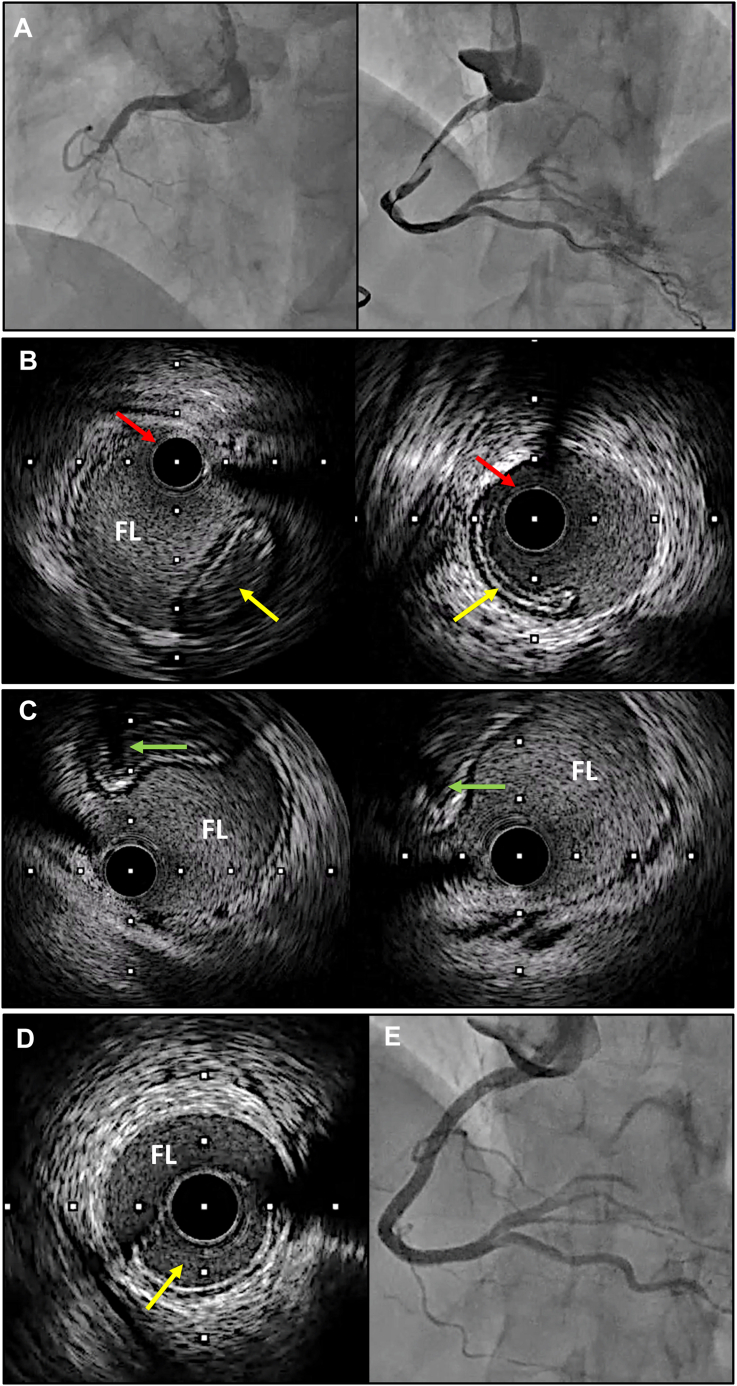
Intravascular ultrasound (IVUS)-guided stenting for iatrogenic aortocoronary dissection. (**A**) Iatrogenic aortocoronary dissection after guiding-catheter engagement into the right coronary artery. (**B**) “Apple turnover–like appearance” of the true lumen (TL; **yellow arrows**) and the first guidewire with the IVUS catheter in the false lumen (FL; **red arrows**). (**C**) The second guidewire (**green arrows**) into the TL at 2 different levels of the right coronary artery, with the posterior shadow cone. (**D**) The IVUS catheter on the second guidewire insert in the TL (**yellow arrow**). (**E**) Final angiogram showing thrombolysis in myocardial infarction (TIMI) 3 coronary blood flow, without extension of the aortic root hematoma.

We first switched the AL0.75 for a 6-F JR4 guiding catheter (Launcher guiding catheter, Medtronic, Minneapolis, MN). Then, the RCA was wired using a SION blue (Asahi Intecc, Irvine, CA) guidewire, and IVUS was performed to confirm whether the wire was in the true or false lumen (Fig. 1B; Video 3 , view video online). The IVUS catheter was then positioned at the ostium of the RCA to guide TL rewiring. After several attempts, using real-time IVUS, a second guidewire, an SUOH 03 (Asahi Intecc), finally entered the TL (Fig. 1C; Video 4 , view video online). IVUS was repeated over this guidewire and confirmed its position in the TL (Fig. 1D; Video 5 , view video online). Subsequently, PCI was performed, as the usual and final angiography demonstrated thrombolysis in myocardial infarction (TIMI) 3 coronary flow (Fig. 1E; Video 6 , view video online). The patient’s clinical course was favourable, with an uneventful hospitalization. Her left ventricular ejection fraction remained largely preserved, with only mild hypokinesia of the inferior wall. A follow-up aortic computed tomography scan at 48 hours confirmed complete healing of the initial aortic root hematoma, with no visible residual dissection.

This case emphasizes the role of intracoronary imaging in guiding TL wiring in the context of extensive dissection, and the relevance of the off-label use of a hydrophilic guidewire initially designed for chronic total occlusion (CTO) procedures.

The first step in such a situation is to switch to a guiding catheter with a different angulation, so as to approach the artery and the dissection with an alternative trajectory and improve the chances of entering the TL. In this case, IVUS was favoured over optical coherence tomography, as the latter would require contrast injection that might have further extended the dissection. Additionally, IVUS can be left in place, allowing real-time assessment of the positioning of the second guidewire to “thread the eye of the needle.” IVUS played a critical role in both determining the stent length and diameter and optimizing the angioplasty result to ensure the best possible outcome.^2^

The off-label use of a hydrophilic CTO guidewire proved to be a key factor in the success of this case. Originally developed for retrograde CTO procedures, wires such as the SUOH 03 and the SION black (Asahi Intecc) provide a high level of procedural success in wiring the TL during IACD. This success is due to their many properties that make them ideal for wiring the TL while minimizing the risk of extension of the dissection.^3^ In this case, the SUOH 03 design (dual-coil, with a soft and flexible tip) offers good maneuverability while maintaining sufficient support along its body, allowing a nontraumatic advancement through challenging anatomies. These characteristics make it particularly effective in complex PCI settings, such as during IACD or retrograde CTO procedures. The adaptability of such guidewires broadens the therapeutic arsenal for interventional cardiologists, emphasizing the importance of being familiar with the full range of equipment available to address high-risk scenarios effectively.

However, if IVUS-guided true-to-true lumen antegrade wiring fails, alternative strategies remain. A small contrast injection through a microcatheter can help clarify the guidewire’s position relative to the dissection plane. Additional options include cutting balloons, a dissection-reentry strategy, or a retrograde approach when suitable collaterals are present.^2^

Overall, this case demonstrates the value of intracoronary imaging in managing high-risk PCI complications, such as IACD. The case also highlights the importance of ensuring that operators are familiar with all available techniques and equipment to successfully manage these situations.

**Novel Teaching Points**

• IVUS is a “game-changer” in the management of complications, such as IACD, providing real-time imaging that enhances procedural success.
• CTO techniques can be effectively repurposed beyond their original scope, offering innovative solutions to tackle specific complications.
• Hydrophilic CTO guidewires are an invaluable asset in managing IACD, pushing the boundaries of interventional strategies.

## Supplementary Material

Video 1 Back-and-forth movements of the 6F AL0.75 guiding catheter into the RCA.

Video 2 IACD after guiding-catheter engagement into the RCA.

Video 3 “*Apple turnover-like appearance*” of the TL and the first guidewire with the IVUS catheter in the FL.

Video 4 The second guidewire into the TL with the posterior shadow cone.

Video 5 The IVUS catheter on the second guidewire into the TL.

Video 6 Final angiogram showing a TIMI 3 coronary blood flow without extension of the aortic root hematoma.

## References

[1] Sanchez-Jimenez E., Levi Y., Roguin A. (2022). Iatrogenic aortocoronary dissection during right coronary artery procedures: a systematic review of the published literature. J Soc Cardiovasc Angiogr Interv.

[2] Harding S.A., Fairley S.L. (2019). Catheter-induced coronary dissection. JACC Case Rep.

[3] Gasparini G.L., Bollati M., Chiarito M. (2023). SUOH 03 guidewire for the management of coronary artery dissection: insights from a multicenter registry. J Interv Cardiol.

